# Ultrasonographic Changes in Brain Hemodynamics in Patients with Parkinson's Disease and Risk Factors for Cerebrovascular Disease: A Pilot Study

**DOI:** 10.1155/2021/1713496

**Published:** 2021-10-05

**Authors:** Rodrigo Tavares Brisson, Rita de Cássia Leite Fernandes, Josevânia Fulgêncio de Lima Arruda, Liene Duarte Silva, Marco Antônio Sales Dantas de Lima, Ana Lucia Zuma Rosso

**Affiliations:** Department of Neurology, Hospital Universitário Clementino Fraga Filho, Federal University of Rio de Janeiro (UFRJ), Rio de Janeiro, Brazil

## Abstract

Recent epidemiological studies have revealed a correlation between atypical features and worse functional outcomes in Parkinson's disease (PD) patients with cerebrovascular disease (CVD). We aimed to evaluate the brain hemodynamics of PD patients with risk factors for CVD using Doppler ultrasonography. In this prospective pilot study, we randomly included 27 outpatients diagnosed with PD. Transcranial color-coded sonography (TCCS) examinations were performed, obtaining measurements of middle cerebral artery mean flow velocities (Vm), the resistance index (RI), and the pulsatility index (PI). The breath-holding index (BHI) was used to assess cerebrovascular reactivity (cVR). Standardized functional scales (UPDRS III, Hoehn & Yahr scale, and MoCA) were administered. The patients were divided into two groups: those with two or more vascular risk factors (PDvasc) and those with fewer than two vascular risk factors (PDnvasc). Patients in the PDvasc group showed higher PI (1.00 vs. 0.85; *p*=0.020), RI (0.59 vs. 0.5; *p*=0.05), H&Y mean (2.4 vs. 1.4; *p*=0.036), higher frequency of altered cVR (90.9% vs. 25.0%; *p*=0.001), and lower BHI (0.46 vs. 1.01; *p*=0.027). We also divided the patients in other two groups: one with patients with classical and another with akinetic-rigid PD clinical type. Patients with the akinetic-rigid type of PD had significantly higher RI (0.60 vs. 0.51; *p*=0.03), PI (0.99 vs. 0.77; *p*=0.03), higher frequency of altered cVR (80% vs. 35%; *p*=0.02), and lower BHI (0.48 vs. 0.96; *p*=0.05) than patients with classic-type PD. We concluded that TCCS displays impaired cerebrovascular reactivity and a more severe disease pattern in Parkinsonian patients with two or more risk factors for CVD and in the akinetic-rigid type. Doppler ultrasonography may be a useful tool in a clinical setting to investigate PD patients.

## 1. Introduction

Parkinson's disease (PD) is a complex neurodegenerative disease first described two centuries ago and still intensively investigated. The degeneration of dopaminergic neurons in the mesencephalic substantia nigra with Lewy body inclusions is the hallmark of this disease. As no biological marker for PD has yet been identified, the disease has been diagnosed based on clinical criteria [1]. Two different clinical phenotypes are known: one in which tremor is the predominant manifestation—classical type—and another with main rigidity—akinetic-rigid type [2].

One of the exclusionary diagnostic criteria for PD is a positive stroke history related temporally to the Parkinsonian syndrome. However, vascular lesions are a common incidental finding in pathologically confirmed PD [3].

The brains of patients with vascular Parkinsonism (VP), that is, without Lewy body pathology, were found to be severely affected by a small-vessel disease (SVD) associated pathological state, in contrast to the findings in age-matched controls with vascular risk factors such as hypertension and heart disease [3]. It remains unknown whether there is an interaction between the pathology associated with the burden of SVD and idiopathic PD.

The importance of cerebrovascular disease (CVD) as a comorbidity in PD patients is a subject of ongoing interest in the field of movement disorders, as it may adversely affect clinical outcome [4, 5]. On the other hand, in clinicopathological studies demonstrating only vascular pathology, a clinical Parkinsonian syndrome is extremely rare [3]. CVD has been linked to functional and cognitive decline in PD [6, 7]. The mechanisms proposed to lie behind this are endothelial changes and altered vascular reactivity, which ultimately lead to hypoperfusion and hypoxia [8]. In this context, some studies have used transcranial color-coded sonography (TCCS) to provide data on the brain hemodynamics of PD patients with associated CVD [9–11].

Altered cerebrovascular reactivity (cVR) detected by transcranial Doppler (TCD) has already been reported in patients with PD [12–14], but none of these studies revealed a correlation of this finding with risk factors for CVD. Against this background, the objectives of this study were to confirm the influence of risk factors for CVD present in Parkinsonian patients on cerebral hemodynamics through TCCS and to determine its relationship with clinical types and severity of PD.

## 2. Materials and Methods

This study took place in the outpatient department of neurology, Hospital Universitário Clementino Fraga Filho, Federal University of Rio de Janeiro, with subjects being recruited randomly from August through December 2020. Patients with PD with an age of onset after 50 years who had been diagnosed by a movement disorders specialist were included. All patients fulfilled the Movement Disorders Society (MDS) Clinical Diagnostic Criteria for Parkinson Disease [15]. Exclusion criteria included a history of intracranial surgery, traumatic brain injury, dementia, stroke, or an inadequate transtemporal window for the TCCS examination. Those patients unable to hold their breath for at least 15 seconds were also excluded.

The patients were divided into two different groups: the first group comprised PD patients with two or more CVD risk factors (hypertension, diabetes, dyslipidemia, heart disease, lung disease, kidney disease, alcoholism, obesity, or smoking)—the PDvasc group—the other group included PD patients with no more than one CVD risk factor—the PDnvasc group. We also divided the patients in other two groups according to clinical presentation: classical or akinetic-rigid type.

The assessment of PD patients regarding disease severity was carried out using the modified scale of Hoehn & Yahr [16] and UPDRS III. Cognitive status was assessed by performing the Montreal Cognitive Assessment Test (MoCA) [17], and the clinical profiles were evaluated using a structured questionnaire (Supplementary Materials).

**Figure 1:**
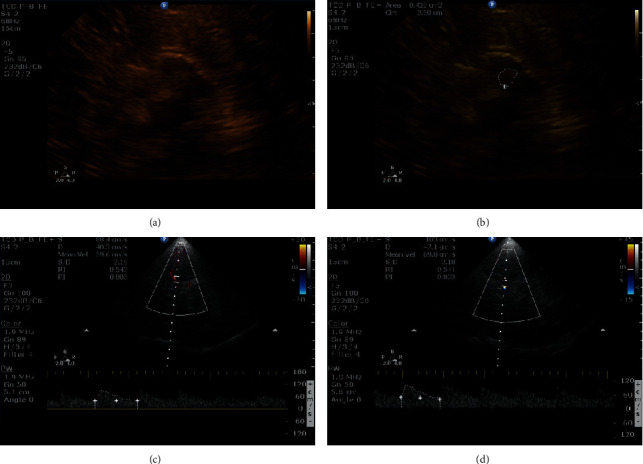
TCCS showing image of the midbrain in (a) and increased SN echogenicity areas (=hyperechogenicity) at the anatomical site of SN in a patient with Parkinson's disease (the area circled in (b)). TCCS examination of intracranial vascular vascularity provided data on the middle cerebral artery (MCA) mean flow velocity (Vm), pulsatility index (PI), and resistance index (RI) of the MCA during rest (c) and after apnea test (“breath-holding test”) (d).

All patients underwent TCCS in the neurosonology laboratory. The TCCS equipment used was an HD11XE (Philips Healthcare, Eindhoven, The Netherlands) with a sectoral transducer of 1–4 MHz. The examination was performed through both temporal windows to obtain standardized axial sections. In the bidimensional mode (B-mode) acquisition, we obtained the substantia nigra echogenic area (cm^2^) (Figure 1). Examination of intracranial vascularity provided data on the mean flow velocity (Vm), pulsatility index (PI), and resistance index (RI) of the middle cerebral artery (MCA) in a resting state (Figure 1). The values obtained from one temporal window were analyzed. Cerebrovascular reactivity was measured using the breath-holding index (BHI) [18].

The BHI is obtained by a test that requires patients to hold their breath for a minimum of 15 seconds and then to restart breathing slowly until returning to the baseline respiratory rate. The BHI is calculated according to the following formula:

(1) $$\begin{array}{c} \text{BHI}=\frac{\text{Vma}-\text{Vmr}}{\text{Vmr}}\times \frac{100}{t},\\ \text{BHI}=\text{breath}-\text{holding index,}\\ \text{Vma}=\text{mean speed after apnea,}\\ \text{Vmr}=\text{mean speed at rest,}\\ t=\text{apnea time in seconds.} \end{array}$$

We considered BHI values <0.69 as abnormal (impaired vasoreactivity), in accordance with previous work [19].

The statistical program SAS Institute Inc 2016, SAS/ACCESS® 9.4, was used for data analysis, computed as the mean ± standard deviation for quantitative variables or frequency (%) for qualitative variables. To determine whether there was a significant difference in the distributions of the quantitative variables between the groups (PDvasc × PDnvasc), Wilcoxon's nonparametric test was performed for independent samples. To confirm which qualitative variables differ between the groups, Fisher's exact test was performed. Statistical significance was set at *p* < 0.05. The study protocol was approved by the local ethics committee, and all patients provided written informed consent for the procedure.

## 3. Results

Twenty-seven PD patients were included in this study, 11 in the PDvasc group and 16 in the PDnvasc group. The clinical and demographic characteristics of the two groups are presented in Table S1. Within the entire cohort, there was a male predominance (88.9%), and the mean age was 64.3 ± 8.7 years. Most individuals had an educational history of longer than 8 years (66.7%). The median disease duration was 5.8 ± 3.3 years. The median H&Y scale score was 2.0, and the median UPDRS III scale score was 18 points. The analysis of cognitive status using MoCA revealed a median in the entire cohort of 22 out of a maximum of 30 points. The mean dose of levodopa was 577.8 mg/day.

Among CVD risk factors, the most common were hypertension (62.9%), smoking (37%), and heart disease (33.3%), followed by diabetes (DM2) (11.1%).

The TCCS disclosed an average substantia nigra size of 0.25 ± 0.14 cm^2^, with no difference between the groups. Doppler analysis revealed a resting Vm value of 44.6 ± 10.3 cm/s, after breath-holding test Vm of 49.37 ± 11 cm/s, PI of 0.85 ± 0.22, RI of 0.55 ± 0.09, and BHI of 0.78 ± 0.68. cVR was found to be altered in 50% of the patients in the entire cohort.

The comparative analysis of ultrasound characteristics and disease severity scales between the two groups showed that individuals in the PDvasc group had higher PI (1.00 vs. 0.75; *p*=0.020), higher RI (0.59 vs. 0.51; *p*=0.05), higher frequency of individuals with altered cVR (90.9% vs. 25%; *p*=0.001), and lower BHI mean values (0.46 vs. 1.01; *p*=0.027) (Table 1). The mean H&Y scale score was significantly higher in individuals in the PDvasc group (2.5 vs. 1.7; *p*=0.036) (Table 2).

Patients with classic-type PD showed lower RI (0.51 vs. 0.60; *p*=0.03), lower PI (0.77 vs. 0.99; *p*=0.03), higher BHI (0.96 vs. 0.48; *p*=0.05), and a lower frequency of individuals with altered cVR (35% vs. 80%; *p*=0.02) than those with the akinetic-rigid phenotype (Table 1).

## 4. Discussion

In this study, we used TCCS to assess the association between brain hemodynamics and the clinical severity of patients with PD, in the presence of risk factors for CVD. We found that individuals in the PDvasc group had lower BHI and higher PI and RI, in addition to a higher rate of changes in cVR, indicating brain vascular pathology. According to Haight et al., changes in cVR are associated with diminished vascular functionality in individuals with hypertension in middle-age and may serve as a preclinical marker for brain dysfunction later in life [20], in agreement with other studies showing that individuals with risk factors for CVD have reduced vascular reactivity across different cortical regions. [21–25].

We also observed that the median H&Y scale score was significantly higher in individuals in the PDvasc group, suggesting a possible influence of vascular risk factors on the clinical severity of PD. These data agree with previous studies that found that patients with PD and CVD risk factors were more prone to rapid deterioration and suffered increased mortality [4, 5, 26]. In a recent study by Malek et al., the presence of two or more vascular risk factors was associated with more rapid deterioration in the UPDRS III scale score, as well as cognitive decline. In addition, if there is a history of stroke or transient ischemic attack, patients with PD are more susceptible to cognitive deterioration than patients without these events (42% vs. 25%) [26].

In some studies, it has already been reported that the rate of progression and the severity of functional disability are lower in individuals with classic-type PD than in those with the akinetic-rigid type. [27] When performing a comparison of the findings of TCCS between the akinetic-rigid type and the classic type, we observed that the mean BHI in patients with the akinetic-rigid type was significantly lower and the RI, PI, and altered cVR were significantly higher. These findings suggest more hemodynamic brain impairment in the akinetic-rigid group and may explain why patients with the classic type have a better prognosis and slower clinical evolution than patients with the akinetic-rigid type of PD.

Xiong et al. described that patients with the akinetic-rigid type have more iron deposition in the globus pallidus than patients with the classic type [28]. Iron deposition in brain tissue has also been reported to be increased in patients with CVD [29], which could explain the differences in vascular Doppler parameters found between these groups.

The TCCS B-mode analysis revealed the typical increase in the echogenic area of the substantia nigra in PD patients. This feature has been exhaustively studied in pursuit of a method for accurately diagnosing PD using ultrasound [30–33]. There was no significant difference in the size of the substantia nigra between the study groups, indicating that this ultrasound marker is related to PD pathology and not to the vascular status of individuals. This appears to be in line with other studies that attributed SN hyperechogenicity to iron deposition in PD-affected brains [34, 35].

To the best of our knowledge, this is the first study in which two groups of PD patients with and without vascular risk factors have been compared using transcranial sonography B-mode imaging of the brain parenchyma and cerebral arterial hemodynamics through Doppler signal acquisition.

Although limited by the small sample size of this pilot study, our results encourage us to replicate this work with a larger number of Parkinsonian patients, as well as to add MRI findings, to obtain a better understanding of the burden of small-vessel cerebrovascular disease in PD patients. The ease of the use of TCCS, with lack of side effects and rapid data acquisition, highlights its usefulness in the clinical setting of outpatient units for investigating PD patients to determine vascular factors that might be responsible for unexpected outcomes of typical patients.

## 5. Conclusion

In this pilot study, we revealed that risk factors for CVD are significantly associated with changes in brain hemodynamics identified by TCCS. Impaired vascular reactivity was also shown to be associated with a worse functional state of patients as quantified by the H&Y scale and with the akinetic-rigid type. Transcranial sonography can guide and complement investigations on brain hemodynamics in PD by providing reliable information on changes in cerebral flow velocity correlated with cerebral activation. Further studies with larger samples are needed to address the influence of vascular risk factors on the pathophysiology of PD, as well as on disease progression, and whether they may have a major impact on the natural course of the disease.

## Figures and Tables

**Table 1:** Comparison between groups of Parkinson's disease phenotypes (akinetic-rigid x classic).

	Akinetic-rigid	Classic	*p* value
Variables	*n* = 10	*n* = 17	
Age (years)	64.5	64.2	0.92
RI	0.6	0.51	**0.03**
PI	0.99	0.77	**0.03**
AI	0.48	0.96	**0.05**
VM after apnea (cm/s)	44.62	52.17	0.19

Altered cVR			
No = AI ≥ 0.70	2 (20)	11 (65)	**0.02**
Yes = AI < 0.69	8 (80)	6 (35)	
Duration of symptoms (years, mean ± SD)	5.60	5.88	0.98
Daily dose of levodopa (mg)	620.0	552.94	0.37

RI: resistance index. PI: pulsatility index. AI: apnea index. cVR: cerebrovascular reactivity.

**Table 2:** Comparison of ultrasound characteristics between the groups (PDvasc x PDnvasc).

	Total sample	PDvasc	PDnvasc	*p*-value
Variable	*n* = 27	*n* = 11	*n* = 16	
Area of SN (cm^2^)	0.25	0.25	0.23	0.656
Vm after apnea (cm/s)	49.37	45.17	52.26	0.126
RI	0.55	0.59	0.51	**0.050**
PI	0.85	1.00	0.75	**0.020**
AI	0.78	0.46	1.01	**0.027**

Altered cVR, *n* (%)				
No = AI ≥ 0.70	13 (48.1)	1 (9.10)	12 (75.0)	**0.001**
Yes = AI < 0.69	14 (51.9)	10 (90.9)	4 (25.0)	
H&Y scale (mean)	2	2.5	1.7	**0.036**
UPDRSIII (median)	18	19	16	0.526

PD phenotype, *n* (%)				
Classic	17 (63)	5 (45.4)	12 (75)	0.223
Akinetic-rigid	10 (37)	6 (54.6)	4 (25)	
MOCA (median)	22	22	22.5	0.676

SN: substantia nigra. RI: resistance index. PI: pulsatility index. AI: apnea index. cVR: cerebrovascular reactivity.

## Data Availability

All data are provided in the Results section of this paper.

## Supplementary Materials

Supplementary Materials Questionnaire on sociodemographic data and on risk factors for cerebrovascular disease. Table S1: clinical and demographic data of the entire sample.

## References

[1] Mhyre T. R., Boyd J. T., Hamill R. W., Maguire-Zeiss K. A. (2012). Parkinson’s disease. *Protein Aggregation and Fibrillogenesis in Cerebral and Systemic Amyloid Disease*.

[2] Prodoehl J., Planetta P. J., Kurani A. S., Comella C. L., Corcos D. M., Vaillancourt D. E. (2013). Differences in brain activation between tremor- and nontremor-dominant Parkinson disease. *JAMA Neurology*.

[3] Zijlmans J. C. M., Katzenschlager R., Daniel S. E., Lees A. J. L. (2004). The L-dopa response in vascular parkinsonism. *Journal of Neurology, Neurosurgery & Psychiatry*.

[4] Gorell J. M., Johnson C. C., Rybicki B. A. (1994). Parkinson’s disease and its comorbid disorders: an analysis of Michigan mortality data, 1970 to 1990. *Neurology*.

[5] Li H. J., Yu Y., Chen Y., Liang H. Y. (2015). Vascular risk factors aggravate the progression of Parkinson’s disease: a five-year follow-up study in Chinese patients. *International Journal of Clinical and Experimental Medicine*.

[6] Chen H., Wan H., Zhang M. (2021). Cerebral small vessel disease may worsen motor function, cognition, and mood in Parkinson’s disease. *Parkinsonism & Related Disorders*.

[7] Ma X., Li S., Li C. (2021). Total cerebral small vessel score association with Hoehn and Yahr stage in Parkinson’s disease. *Frontiers in Aging Neuroscience*.

[8] Paolini Paoletti F., Simoni S., Parnetti L., Gaetani L. (2021). The contribution of small vessel disease to neurodegeneration: focus on Alzheimer’s disease, Parkinson’s disease and multiple sclerosis. *International Journal of Molecular Sciences*.

[9] Tsai C.-F., Wu R.-M., Huang Y.-W., Chen L.-L., Yip P.-K., Jeng J.-S. (2007). Transcranial color-coded sonography helps differentiation between idiopathic Parkinson’s disease and vascular parkinsonism. *Journal of Neurology*.

[10] Venegas-Francke P. (2010). Transcranial sonography in the discrimination of Parkinson’s disease versus vascular parkinsonism. *International Review of Neurobiology*.

[11] Caba L. M., Ferrairó J. I. T., Torres I. M., Costa J. F. V., Muñoz R. B., Martin A. L. (2020). El índice de pulsatilidad intracraneal elevado apoya el diagnóstico de parkinsonismo vascular frente a enfermedad de Parkinson idiopática. *Neurologia*.

[12] Vokatch N., Grötzsch H., Mermillod B., Burkhard P. R., Sztajzel R. (2007). Is cerebral autoregulation impaired in Parkinson’s disease? A transcranial Doppler study. *Journal of Neurological Sciences*.

[13] Camargo C. H. F. (2015). Abnormal cerebrovascular reactivity in patients with Parkinson’s disease. *Parkinson’s Disease,*.

[14] Ojeda A. E., Martinez H. R., Rivera F. G., Escamilla Garza J. M., Medina H. C., Davila S. S. (2017). Cerebral vasoreactivity in Parkinson’s disease: a cross-sectional pilot study in a Hispanic cohort. *Journal of Alzheimers Disease and Parkinsonism*.

[15] Postuma R. B., Berg D., Stern M. (2015). MDS clinical diagnostic criteria for Parkinson’s disease. *Movement Disorders*.

[16] Goetz C. G., Poewe W., Rascol O. (2004). Movement disorder society task force report on the Hoehn and Yahr staging scale: status and recommendations the movement disorder society task force on rating scales for Parkinson’s disease. *Movement Disorders*.

[17] Nasreddine Z. S., Phillips N. A., Badirian V. r. (2005). The montreal cognitive assessment, MoCA: a brief screening tool for mild cognitive impairment. *Journal of the American Geriatrics Society*.

[18] Markus H. S., Harrison M. J. (1992). Estimation of cerebrovascular reactivity using transcranial Doppler, including the use of breath-holding as the vasodilatory stimulus. *Stroke*.

[19] Vernieri F., Pasqualetti P., Passarelli F., Rossini P. M., Silvestrini M. (1999). Outcome of carotid artery occlusion is predicted by cerebrovascular reactivity. *Stroke*.

[20] Haight T. J., Bryan R. N., Erus G. (2015). Vascular risk factors, cerebrovascular reactivity, and the default-mode brain network. *NeuroImage*.

[21] Claus J. J., Breteler M. M. B., Hasan D. (1998). Regional cerebral blood flow and cerebrovascular risk factors in the elderly population. *Neurobiology of Aging*.

[22] Riecker A., Grodd W., Klose U. (2003). Relation between regional functional MRI activation and vascular reactivity to carbon dioxide during normal aging. *Journal of Cerebral Blood Flow and Metabolism*.

[23] Jennings J. R., Muldoon M. F., Ryan C. (2005). Reduced cerebral blood flow response and compensation among patients with untreated hypertension. *Neurology*.

[24] Last D., Alsop D. C., Abduljalil A. M. (2007). Global and regional effects of type 2 diabetes on brain tissue volumes and cerebral vasoreactivity. *Diabetes Care*.

[25] Glodzik L., Rusinek H., Brys M. (2011). Framingham cardiovascular risk profile correlates with impaired hippocampal and cortical vasoreactivity to hypercapnia. *Journal of Cerebral Blood Flow and Metabolism*.

[26] Malek N., Lawton M. A., Swallow D. M. A. (2016). Vascular disease and vascular risk factors in relation to motor features and cognition in early Parkinson’s disease. *Movement Disorders*.

[27] Kalia L. V., Lang A. E. (2015). Parkinson’s disease. *The Lancet*.

[28] Xiong W., Li L.-F., Huang L. (2020). Different iron deposition patterns in akinetic/rigid-dominant and tremor-dominant Parkinson’s disease. *Clinical Neurology and Neurosurgery*.

[29] Liu Y., Liu J., Liu H. (2016). Investigation of cerebral iron deposition in aged patients with ischemic cerebrovascular disease using susceptibility-weighted imaging. *Therapeutics and Clinical Risk Management*.

[30] Vlaar A. M. M., Bouwmans A., Mess W. H., Tromp S. C., Weber W. E. J. (2009). Transcranial duplex in the differential diagnosis of parkinsonian syndromes. *Journal of Neurology*.

[31] Berg D. (2011). Enlarged substantia nigra hyperechogenicity and risk for Parkinson disease. *Archives of Neurology*.

[32] Fernandes R. C., Berg D. (2015). Parenchymal imaging in movement disorders. *Frontiers of neurology and neuroscience*.

[33] Li D.-H., He Y.-C., Liu J., Chen S.-D. (2016). Diagnostic accuracy of transcranial sonography of the substantia nigra in Parkinson’s disease: a systematic review and meta-analysis. *Scientific Reports*.

[34] Berg D., Roggendorf W., Schröder U. (2002). Echogenicity of the substantia nigra. *Archives of Neurology*.

[35] Zecca L., Berg D., Arzberger T. (2005). In vivo detection of iron and neuromelanin by transcranial sonography: a new approach for early detection of substantia nigra damage. *Movement Disorders*.

