# Supplementary material for: Ultrasonographic Changes in Brain Hemodynamics in Patients with Parkinson's Disease and Risk Factors for Cerebrovascular Disease: A Pilot Study
Source: Parkinsons Dis. 2021 Oct 5;2021:1713496. doi: 10.1155/2021/1713496 (PMC8510819; doi:10.1155/2021/1713496)
Supplement: Supplementary Materials — Questionnaire on sociodemographic data and on risk factors for cerebrovascular disease. Table S1: clinical and demographic data of the entire sample. [file 1713496.f1.docx]

**Supplementary Materials**

Questionnaire on sociodemographic data and on risk factors for cerebrovascular disease

Name: Date:

Hospital identification number: Date of birth: Age:

Educational history ( ) < 8 years ( ) > 8 years

Ethnicity ( )Caucasian ( ) Other

Preponderant laterality ( ) right ( ) left

Family history of PD ( ) yes ( ) no

Depression ( ) yes ( ) no

Smoking ( ) yes ( ) no

Alcoholism ( ) yes ( ) no

Atrial fibrillation ( ) yes ( ) no

Chronic kidney failure ( ) yes ( ) no

Congestive heart failure ( ) yes ( ) no

COPD ( ) yes ( ) no

Coronary heart disease ( ) yes ( ) no

Type II diabetes ( ) yes ( ) no

Hyperlipidemia ( ) yes ( ) no

Systemic arterial hypertension ( ) yes ( ) no

Obstructive sleep apnea ( ) yes ( ) no

Peripheral vascular disease ( ) yes ( ) no

Stroke ( ) yes ( ) no

Valvular heart disease ( ) yes ( ) no

Date of onset of symptoms (rigidity, tremor, bradykinesia, etc.) and segment initially affected:

Medicines in use:

Daily dose of L-dopa:

Duration of illness:

| **Table S1 - Clinical and demographic data** | | | | |
| --- | --- | --- | --- | --- |
| **Variable** | **Total sample** | **PDvasc** | **PDnvasc** | ***p-value*** |
|  | **n=27** | **n=11** | **n=16** |  |
| **Sex, n(%)** |  |  |  |  |
| *Male* | 24(88.9) | 9(81.8) | 15(93.7) |  |
| *Female* | 3 (11.1) | 2(18.2) | 1(6.3) |  |
| **Age (years;mean ± SD)** | 64.3 ± 8.7 | 69.36 ± 6.7 | 60.81 ± 8.3 | **0.004** |
| **Ethnicity** |  |  |  |  |
| *Caucasian* | 17 (62.9) | 7 (63.5) | 10(62.5) | 1.00 |
| *Other* | 10 (37.1) | 4 (36.4) | 6 (537.5) |  |
| **Educational history (years), n (%)** |  |  |  |  |
| *<8 years* | 9 (33.3) | 3 (27.7) | 6 (37.5) | 0.579 |
| *>8 years* | 18 (66.7) | 8 (72.7) | 10 (62.5) |  |
| **Disease duration (years), n (%)** |  |  |  |  |
| *2–5 years* | 14 (51.9) | 5 (45.5) | 9 (56.3) | 0.317 |
| *5–10 years* | 9 (33.3) | 3 (27.3) | 6 (37.5) |  |
| *More than 10 years* | 4 (14.8) | 3 (27.3) | 1 (6.25) |  |
| **Initial clinical manifestation** |  |  |  |  |
| *Tremor* | 17 (63) | 6 (54.6) | 11 (68.8) | 0.687 |
| *Rigidity/bradykinesia* | 10 (37) | 5 (45.4) | 5 (31.2) |  |
| **Predominant laterality, n (%)** |  |  |  |  |
| *Right* | 19 (70.4) | 7 (63.6) | 12 (75) | 0.675 |
| *Left* | 8 (29.6) | 4 (36.4) | 4 (25) |  |
| **Smoker, n (%)** | 10 (37) | 2 (18,2) | 6 (37.5) | 0.280 |
| **DM2, n(%)** | 3 (11.1) | 3 (27.3) | 0 (0) | **0.050** |
| **Depression, n (%)** | 7 (25.9) | 2 (18.2) | 5 (31.25) | 0.661 |
| **Caffeine consumption, n (%)** | 23 (85.2) | 10 (90.9) | 13 (81.25) | 0.623 |
| **Stroke, n (%)** | 0 (0.0) | 0 (0) | 0 (0) |  |
| **SAH, n (%)** | 17 (62.9) | 11 (100) | 6 (37.5) | **0.001** |
| **Dyslipidemia, n (%)** | 5 (18.5) | 5 (45.5) | 0 (0) | **0.005** |
| **Cardiac disease, n (%)** | 9 (33.3) | 8 (72.7) | 1 (6.25) | **0.003** |
| **Lung disease, n (%)** | 2 (7.41) | 1 (9.0) | 1 (6.25) | 1.000 |
| **Alcoholism, n (%)** | 6 (22.2) | 2 (18.2) | 4 (25.0) | 1.000 |
| **Duration of symptoms (years, mean ± SD)** | 5.8 (3.3) | 6.82 (4.25) | 5.06 (2.11) | 0.353 |
| **Daily dose of levodopa (mg)** | 577.8 | 624.55 | 525 | 0.638 |

- SAH: systemic arterial hypertension
- DM2: type 2 diabetes mellitus
- SD: standard deviation
- PDvasc: Patients with two or more vascular risk factors
- PDnvasc: Patients with fewer than two vascular risk factors
